# Does Trunk Self-Elongation Instruction Lead to Changes in Effective Trunk Height and Spino-Pelvic Parameters? A Radiographic Analysis

**DOI:** 10.3390/jfmk9040253

**Published:** 2024-12-03

**Authors:** Grégoire Prum, Camille Eyssartier, Maxime Bourgain, Philippe Rouch, Pierre Billard, Patricia Thoreux, Christophe Sauret

**Affiliations:** 1 Institut de Biomécanique Humaine Georges Charpak, Arts et Métiers Sciences and Technologies, 75013 Paris, France; gregoire.prum@chu-rouen.fr (G.P.); maxime.bourgain@epf.fr (M.B.);; 2 Unité de Médecine Physique et Rééducation, Rouen University Hospital, 76000 Rouen, France; 3 French Gymnastics Federation, 75010 Paris, France; 4 EPF School of Engineering, 94230 Cachan, France; 5 Grand Hôpital de l’Est Francilien, 77100 Meaux, France; 6 Institut de Biomécanique Humaine Georges Charpak, Université Sorbonne Paris Nord, 93430 Villetaneuse, France; 7 Centre d’Investigations en Médecine du Sport, Hôpital Hôtel Dieu, Assistance Publique-Hôpitaux de Paris, 75004 Paris, France; 8 Center for Research and Studies on Assistive Devices for People with Disabilities (CERAH), Institution Nationale des Invalides, 75007 Paris, France

**Keywords:** rehabilitation, core strengthening, thoracic kyphosis, lumbar lordosis, gymnastics

## Abstract

Background/Objectives: The aim of this study was to evaluate changes in trunk height and variations in spino-pelvic parameters during trunk self-elongation. Two populations were studied: non-athletes and gymnasts, who differ in their engagement with core-strengthening exercises. Methods: EOS biplanar radiographs were taken on 14 non-athletes and 24 gymnasts in both neutral and trunk self-elongation positions. Three-dimensional reconstructions of the pelvis and spine were used to calculate effective trunk height, thoracic and lumbar contributions, and spino-pelvic parameters. Results: Trunk self-elongation resulted in a significant increase in trunk height for both groups (7 mm on average, range: −1 to 14 mm), accompanied by a reduction in thoracic kyphosis for all participants (−10° for non-athletes and −17° for gymnasts, on average) and a reduction in lumbar lordosis in most participants (−5° for non-athletes and −7° for gymnasts, on average). However, some individuals in both groups exhibited an increase in lumbar lordosis, which reduced the contribution of the lumbar region to overall trunk height. Conclusions: Trunk self-elongation instruction effectively increases trunk height, but additional instructions, such as pelvic retroversion, may enhance its effectiveness.

## 1. Introduction

Active axial extension of the spine, also referred to as trunk self-correction or trunk self-elongation, is a common exercise used in prevention, rehabilitation, physiotherapy, and core training for athletes across various disciplines. Its primary aim is to activate the trunk’s postural muscles, resulting in a reduction in spinal curvatures—namely lumbar lordosis, thoracic kyphosis, and cervical lordosis—which leads to an increase in effective trunk height during the exercise. Regular practice of this exercise is believed to strengthen the postural muscles of the trunk, potentially playing a protective role for the spine. In rehabilitation, this exercise is applied in specific pathological conditions, such as improving postural control in adolescents with idiopathic scoliosis [1,2,3], in patients with multiple sclerosis [4], or in reducing forward trunk flexion in Parkinson disease [5]. It is also widely recommended by coaches in sport training, particularly in gymnastics, Pilates, and yoga (as in the Tadāsana posture) to enhance activation of the deep trunk muscles.

To guide the patient or athlete in performing this exercise, the most commonly used instruction is “Straighten your back” [4,6,7,8], although other cues like ”Stand up straight” or ”Push the skull toward the sky” may also be used. The goals of axial spine extension differ between the general population and the athletic population, as do its effects on the spine. For the former, the objective is often to correct postural disorders (particularly excessive thoracic kyphosis resulting from prolonged sitting habits), while for athletes, the aim is to enhance performance and prevent injuries.

Spino-pelvic balance relies on the harmonious distribution of spinal curves and pelvic parameters, which are unique to each individual [9,10]. This balance is influenced by morphological factors, such as pelvic incidence, and static factors, such as sacral slope [11]. Various methods have been used to study the effect of active trunk axial extension on spino-pelvic balance, including raster stereography (i.e., Moiré’s technique) [6,12], Saunders inclinometer [7,8], surface topography and biplanar radiographs [1], optoelectronic systems [13,14], and photogrammetry images [15]. However, measuring axial extension in terms of trunk height variation remains challenging due to difficulties in quantifying this change using external markers or the body’s external shape. Therefore, 3D reconstructions of bones from medical images, such as the EOS^®^ stereoradiographic system [16], could provide valuable insights. This system captures simultaneous frontal and lateral radiographic images of subjects in a standing posture with low-dose X-ray exposure, enabling 3D reconstruction of the pelvis and the spine [17,18,19,20,21], which are widely used in clinical practice. This technology offers a potential method for assessing trunk height changes and their distribution between the lumbar and thoracic regions during trunk axial extension. Moreover, unlike measurement on sagittal radiographs, this technique enables access to 3D space, eliminating projection errors in the quantification of parameters.

The accuracy of measurements from EOS images ranges from 3° to 6° for kyphosis and lordosis calculations and from 1° to 3° for pelvic balance parameters [19,21]. This system and its associated 3D reconstruction methods can confidently evaluate the effect of trunk self-elongation instruction on spine height (lumbar spine height, thoracic height, and total trunk height) and spino-pelvic parameters (lumbar lordosis, thoracic kyphosis, sacral slope, and pelvic tilt).

Given the lack of validated data regarding effective change in trunk height during trunk self-elongation, this study aims to measure the effect of this exercise on spine curvatures and trunk height using EOS biplanar radiographs. It is hypothesized that active axial extension results in a measurable increase in the trunk height enabled by a change in spino-pelvic balance. Additionally, since the changes are expected to result from muscle contractions, it is hypothesized that they will be more pronounced in athletes, such as gymnasts, who regularly practice spine straightening in a performance objective.

## 2. Materials and Methods

### 2.1. Participants

After obtaining ethical approval for this study (ID RCP: 2018-A01926-49), 14 healthy individuals from the general population (6 males and 8 females; mean age: 32.1 ± 13.8 years old) and 24 gymnasts (9 males and 15 females; mean age: 14.5 ± 2.4 years old) volunteered to take part in this study. The gymnasts trained for more than fifteen hours per week and competed at least at the national level in artistic or rhythmic gymnastics. Group characteristics are reported in Table 1.

Inclusion criteria for the general population group (referred to as the non-athlete group) were being over 18 years of age and reporting no more than moderate activity soliciting the trunk muscles. For gymnasts, inclusion criteria were being over 12 years of age and having no history of spine or pelvis surgery. Pregnancy was an exclusion criterion for both groups.

To avoid learning bias, instructions were provided to participants a few days before the acquisition session. They were required to familiarize themselves with the instructions under the supervision of a sport instructor, but no specific guidance was given regarding how to achieve and maintain the trunk self-elongation posture.

### 2.2. Procedures

Each subject underwent a low-dose biplanar radiograph (face and lateral views) using the EOS system (EOS imaging, Paris, France) [16] in both a standardized neutral standing position (N) and during trunk self-elongation (S). The instructions given to participants were “Take your usual standing position looking forward” for the neutral position and “Straighten your back and try to push the top of your skull towards the sky” for the trunk self-elongation condition to maximize spinal straightening. The EOS acquisition lasted approximately 20 s, during which the participants were instructed to remain as still as possible. The biplanar radiographs enabled 3D reconstructions of the pelvic bones and vertebrae [17,18,19,20,21], which were performed by one operator and subsequently verified by a second operator, both trained and certified for this process. To calculate pelvic and spinal parameters, a regionalization of each bone of interest (T1, T4, T9, T12, L1, and L5 vertebrae and pelvis) was carried out based on these 3D reconstructions. Static and morphological parameters of the pelvis and spine were then calculated (see Table 2 for definitions).

### 2.3. Data Processing and Analyses

**Figure 1:**
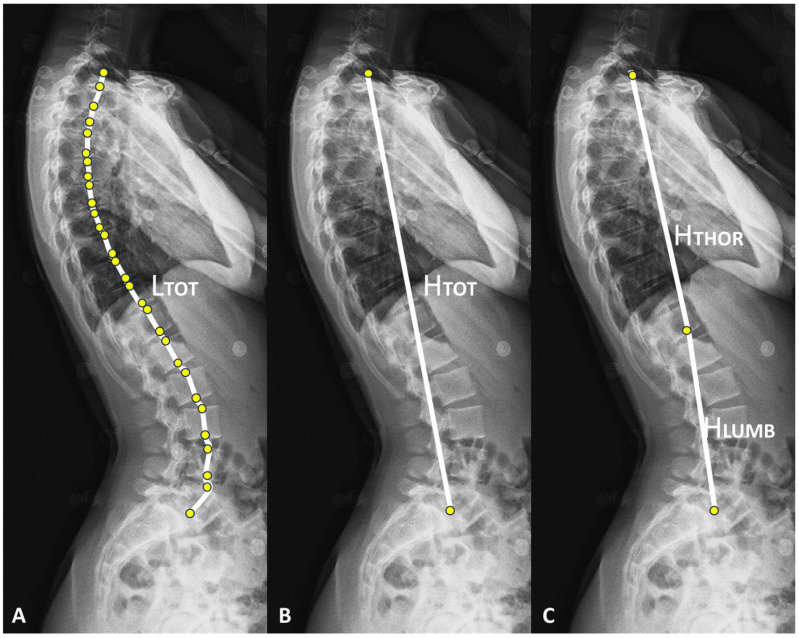
(**A**–**C**) Illustrations on a lateral EOS radiograph of the length of the thoracolumbar spine (L_TOT_), the height of the thoracolumbar spine (H_TOT_), the height of the thoracic spine (H_THOR_), and the height of the lumbar spine (H_LUMB_).

Variations in thoracolumbar spine height (ΔH_TOT_), thoracic spine height (ΔH_THOR_), and lumbar spine height (ΔH_LUMB_) between the two acquisitions were computed in millimeters (mm) from the 3D reconstructions. To avoid the bias due to the participant height in ΔH_TOT_, ΔH_THOR_, and ΔH_LUMB_, their respective ratios (R_TOT_, R_THOR_, R_LUMB_, in %) relative to the length of the thoracolumbar spine (L_TOT_) were also calculated (Figure 1). Additionally, changes in the following parameters during the trunk self-elongation posture were analyzed: variations in sacral slope (ΔSS), pelvic tilt (ΔPT), thoracic kyphosis (ΔTK), T4T12 kyphosis (ΔTK4-12), and lumbar lordosis (ΔLL), all expressed in degrees. As pelvic incidence (PI) and thoracolumbar spine (L_TOT_) are morphological parameters and therefore are not supposed to change between the two postures, they were calculated based on the neutral acquisition (N).

### 2.4. Statistical Analyses

Spino-pelvic parameters in neutral (N) and trunk self-extension conditions (S) were compared using a Wilcoxon signed rank exact test for paired data within each group. Statistical tests were performed using R software (version 4.3.3) with a significance level of 5%, and *p*-values are reported.

### 2.5. Use of Generative AI Tools

In preparing this article, the authors used DeepL (https://www.deepl.com) and ChatGPT (https://chatgpt.com/) for final proofreading prior to submission. These tools were used with instructions to identify and correct grammatical and spelling errors in English, as well as to occasionally refine sentence structure for clarity. All suggested changes were thoroughly reviewed and, when necessary, adjusted to preserve the original meaning. As such, the generative AI tools did not contribute to the scientific content of this article.

## 3. Results

Spino-pelvic parameters in both the neutral and trunk self-elongation positions for the two groups are presented in Table 3. Comparison between the two positions revealed significant changes in thoracic parameters for both groups, with an increase in H_THOR_ and a decrease in thoracic kyphosis (TK and TK4-12). Lumbar lordosis also significantly decreased in both groups, though this reduction was less pronounced in non-athletes compared to gymnasts, leading to a significant difference in H_LUMB_ only for gymnasts. Regarding the pelvic parameters, significant differences between the two positions were observed only in gymnasts, with a decrease in SS and an increase in PT. Lastly, total trunk height (H_TOT_) increased significantly between the two positions for both groups.

Specifically, the trunk self-elongation instruction led to an average increase in H_TOT_ of 7.6 ± 3.6 mm (range: 1.9 to 13.4 mm) for non-athletes; and of 7.1 ± 3.7 mm (range: −0.5 to 13.7 mm) in gymnasts. Overall, all participants showed an increased in H_TOT_, except for two female gymnasts whose H_TOT_ decreased slightly (range: −0.5 and −0.3 mm), which remains within the margin of measurement accuracy.

**Figure 2:**
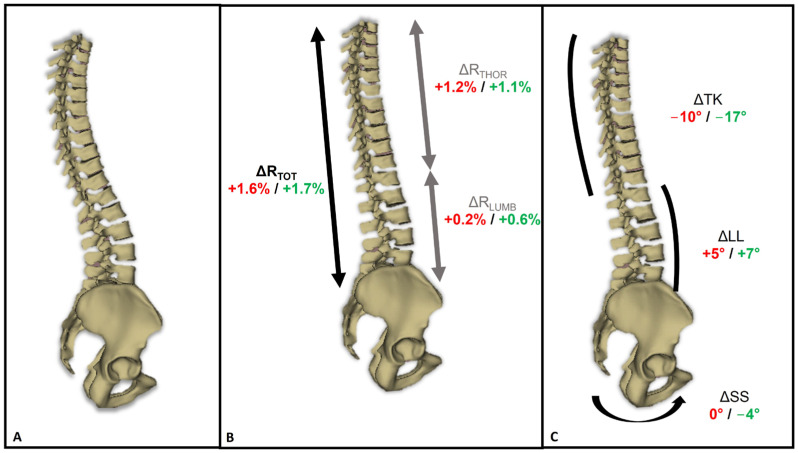
(**A**) Example of a 3D reconstruction of the thoracolumbar spine and pelvis in the neutral position; (**B**,**C**) 3D reconstruction of the spine and pelvis during the trunk self-elongation position, with average changes in the (**B**) ratio of thoracolumbar spine height (ΔR_TOT_), thoracic spine height (ΔR_THOR_), and lumbar spine height (ΔR_LUMB_); and (**C**) sacral slope (ΔSS), lumbar lordosis (ΔLL), and thoracic kyphosis (ΔTK). Font color: red for non-athletes; green for gymnasts.

Regarding the respective contributions of the lumbar and thoracic regions to axial spine extension, both the lumbar and thoracic spine heights increased, albeit to different extents. In non-athletes, ΔH_THOR_ increased by 5.8 ± 2.8 mm (range: 1.8 to 9.3 mm), and ΔH_LUMB_ increased by 0.9 ± 1.8 mm (range: −2.0 to 5.4 mm), resulting in spine straightening ratios of 1.3 ± 0.6% for R_THOR_ (range: 0.4 to 2.1%), 0.2 ± 0.4% for R_LUMB_ (range: −0.5 to 1.2%), and 1.7 ± 0.8% for R_TOT_ (range: 0.5 to 3.0%) (Table 4). In gymnasts, ΔH_THOR_ increased by 4.7 ± 2.6 mm (range: 0.1 to 9.2 cm) and ΔH_LUMB_ increased by 2.5 ± 2.4 mm (range: −2.2 to 6.9 mm), resulting in spine straightening ratios of 1.2 ± 0.7% for R_THOR_ (range: 0.0 to 2.4%), 0.7 ± 0.6% for R_LUMB_ (range: −0.6 to 1.7%), and 1.8 ± 1.0% for R_TOT_ (range: −0.1% to 3.4%) (Table 4). None of the participants in either group showed a decrease in thoracic spine height, while four gymnasts (three men and one woman) and five non-athletes (three men and two women) showed a decrease in the lumbar spine height ratio. Figure 2 presents a visual representation of the main results of this study.

## 4. Discussion

Trunk self-elongation is an exercise believed to strengthen core muscles, which would kinematically lead to an increase in trunk height during execution. This increase would be enabled by a reduction in spinal curvatures, specifically decreases in both lumbar lordosis and thoracic kyphosis. A reduction in lumbar lordosis is also expected to be associated with a decrease in sacral slope accompanied by an increase in pelvic tilt [22]. Two populations were studied, and it was hypothesized that gymnasts, who undergo intensive core strength training and frequently perform exercises that reinforce their back muscles, would exhibit more pronounced changes in spino-pelvic parameters and trunk height compared to non-athletes.

The novelty and methodological strength of this study lie in the use of radiographic imaging combined with 3D reconstruction methods to assess spino-pelvic parameters and trunk height. Unlike studies that rely on external measuring devices prone to soft tissue artifacts and palpation errors [23], such as inclinometers [8,24] or optoelectronic motion capture systems paired or not paired with computational models [14,25], the use of the EOS system provides reliable direct 3D measurements. Furthermore, in this study, spine height was not based on projection onto the vertical axis but was calculated as the actual distance between the relevant vertebrae in the 3D space. This methodological approach allows for accurate quantification of spinal height gains even in cases of spinal balance disturbances (e.g., scoliosis). Additionally, the measurements enabled an assessment of the relative contributions of the lumbar and thoracic spine to overall straightening.

Apart from the age and anthropometric differences between the two groups, there were also baseline differences in spino-pelvic parameters. In particular, pelvic tilt, pelvic incidence, and thoracic kyphosis were lower in gymnasts compared to non-athletes, likely due to postural training in gymnastics and the regular use of thoracolumbar straightening exercises. Previous literature has reported changes in spinal curvatures among athletes engaged in various sports: inline hockey, freestyle wrestling, and cycling tend to increase thoracic kyphosis, while handball tends to decrease lumbar lordosis [26,27,28,29]. However, comparisons of spino-pelvic parameters between the groups were not the objective of this study, so no statistical analysis was performed for this purpose.

Regarding the effect of trunk self-elongation compared to the neutral position, this study demonstrated that both populations were able to increase their trunk height, with gymnasts showing a slightly greater increase than non-athletes (Table 4). However, the contribution of the thoracic region was slightly lower in gymnasts than in non-athletes, likely due to the lower thoracic kyphosis in gymnasts, which limits further straightening potential. In the lumbar region, the greater reduction in lumbar lordosis in gymnasts, driven by significant changes in pelvic positional parameters (i.e., decreased sacral slope and increased pelvic tilt), contributed to a greater increase in trunk height (through an increase in R_LUMB_). The changes observed in non-athletes support the potential benefit of this exercise in rehabilitation, as strengthening the thoracic muscles could help correct the excessive thoracic kyphosis commonly seen in sedentary individuals with prolonged sitting habits [30]. Findings from D’Amico et al. also align with these results, showing a significant decrease in thoracic kyphosis in men, without notable changes in lumbar lordosis [13].

For the lumbar region, this study highlights the value of trunk self-elongation, which, through voluntary actions, reduces lumbar lordosis in most participants, both non-athletes and gymnasts, potentially preventing hyper-lordosis-related pathologies. However, it is important to note that for some participants (five non-athletes and four gymnasts; representing 36% of non-athletes and 16% of gymnasts), R_LUMB_ decreased during the trunk self-elongation exercise, which could increase the stress on the posterior vertebral arch of the lumbar spine, contrary to the intended outcome. Since both non-athlete and gymnast participants exhibited this behavior, the strength deficit of lumbar core muscles may not be the only factor, and additional instructions could be helpful. Specifically, it could be hypothesized that adding an instruction of pelvic retroversion (reducing sacral slope) along with the “straightening” instruction may help decrease lumbar lordosis and address this trend in all subjects. However, this requires further evaluation.

### Limitation

Although this study allowed for the evaluation of the impact of active axial spine extension on trunk height and spino-pelvic balance with improved reliability compared to previous methods, it is not free of limitations. First, while instructions were sent to participants prior to the data acquisition session so they could practice the posture, there may still be bias due to participants’ interpretation of the instructions. As a result, the findings may partly reflect the participants’ understanding of the instructions rather than their full ability to elongate their trunk, though this was true for both populations. Additionally, although both non-athletes and gymnasts were studied, comparing these two groups should be carried out with caution, as they differed in more than just physical activity; age, height, and weight were also variables. The authors emphasize that the primary objective of this study was not to compare these two populations, but rather to compare two postures (with vs. without trunk self-elongation instruction) and to highlight the different strategies each group adopted in achieving trunk elongation. Finally, although a larger cohort could have strengthened the statistical analysis, the current sample size is sufficient to address this study’s hypothesis. Hence, significantly increasing the sample size might raise ethical concerns.

## 5. Conclusions

This study hypothesized that trunk self-elongation would lead to an effective increase in trunk height, achieved through changes in spino-pelvic balance. This hypothesis was tested in two populations with differing levels of physical activity. Overall, the instruction effectively resulted in an increase in trunk height, primarily through the correcting of thoracic kyphosis and a reduction in lumbar lordosis in both groups, though the contribution of the lumbar region varied between participants from the two populations.

Given that the observed changes were likely due to muscle contractions, it was further hypothesized that a more pronounced effect would be seen in gymnasts, who regularly practice spine-strengthening exercises as part of their training. The results showed that the increase in total trunk height for non-athletes was primarily due to an increase in thoracic height, while in gymnasts, the straightening effect was more evenly distributed between the thoracic and lumbar spine. Therefore, it would be interesting to evaluate whether regular training with such exercises could enhance the contribution of the lumbar spine in non-athletes. However, in some individuals, from both non-athletes and gymnasts, the trunk self-elongation instruction unexpectedly led to an increase in lumbar lordosis.

Finally, this study confirms the expected effects of this exercise, which is widely used in both clinical practice and sports training, for activating and strengthening deep trunk muscles, making it valuable for the prevention and treatment of back pain [31]. However, additional instruction on pelvic retroversion, for instance, may be beneficial for some subjects to ensure lumbar contribution without being detrimental for other subjects. The effectiveness of such additional instruction, however, remains to be evaluated.

## Figures and Tables

**Table 1:** Participants characteristics for non-athlete and gymnast groups (mean ± SD).

Characteristics	Non-Athlete	Gymnast
Age (years)	32.1 ± 13.8	14.5 ± 2.4
Weight (kg)	67.0 ± 16.1	48.6 ± 12.3
Height (m)	1.65 ± 0.08	1.55 ± 0.09

**Table 2:** Pelvic and spinal parameters’ definitions. L_TOT_, H_TOT_, H_THOR_, and H_LUMB_ are spinal morphological parameters; TK, TK4-12, and LL are spinal balance parameters, and PI, SS, and PT are pelvic parameters.

Parameters (Unit)	Abbreviation	Definition
Length of the thoracolumbar spine (mm)	L_TOT_	Length of the broken line crossing each center of vertebral endplates from the cranial endplate of the first thoracic vertebra (T1) to the caudal endplate of the fifth lumbar vertebra (L5).
Thoracolumbar spine height (mm)	H_TOT_	Distance between the centers of the cranial endplate of first thoracic vertebra (T1) and the caudal endplate of fifth lumbar vertebra (L5).
Thoracic spine height (mm)	H_THOR_	Distance between the centers of the cranial endplate of the first thoracic vertebra (T1) and the caudal endplate of the twelfth thoracic vertebra (T12).
Lumbar spine height (mm)	H_LUMB_	Distance between the centers of the caudal endplate of twelfth thoracic vertebra (T12) and the caudal endplate of the fifth lumbar vertebra (L5).
Thoracic kyphosis (°)	TK	Angle between the cranial endplate of the first thoracic vertebra (T1) and the caudal endplate of the twelfth thoracic vertebra (T12).
T4-T12 thoracic kyphosis (°)	TK4-12	Angle between the cranial endplate of fourth thoracic vertebra (T4) and the caudal endplate of the twelfth thoracic vertebra (T12).
Lumbar lordosis (°)	LL	Angle between the cranial endplate of the first lumbar vertebra (L1) and the caudal endplate of fifth lumbar vertebra (L5).
Pelvic incidence (°)	PI	Angle between the line connecting the sacral endplate midpoint to the hip axis and the perpendicular axis to the sacral plate crossing this point.
Sacral slope (°)	SS	Angle between the horizontal axis and the superior endplate of first sacral vertebra (S1), in the sagittal plane.
Pelvic tilt (°)	PT	Angle between the vertical and the axis crossing the sacral endplate midpoint to the hip axis, in the sagittal plane.

**Table 3:** The spino-pelvic parameters in both neutral (N) and trunk self-elongation (S) positions. The *p*-value for every comparison is reported for both non-athlete and gymnast groups. Significant differences are highlighted using bold font.

Parameters (Unit)	Non-Athletes			Gymnasts		
	N	S	*p*	N	S	*p*
L_TOT_ (mm)	458 ± 20	-	-	403 ± 33	-	-
H_TOT_ (mm)	432 ± 16	439 ± 19	**<0.001**	384 ± 32	390 ± 34	**<0.001**
H_THOR_ (mm)	264 ± 13	270 ± 13	**<0.001**	236 ± 19	241 ± 20	**<0.001**
H_LUMB_ (mm)	194 ± 9	195 ± 9	0.06	168 ± 17	171 ± 18	**<0.001**
TK (°)	41 ± 11	31 ± 11	**<0.001**	33 ± 10	16 ± 15	**<0.001**
TK4-12 (°)	38 ± 9	30 ± 9	**0.001**	33 ± 9	20 ± 13	**<0.001**
LL (°)	−33 ± 13	−28 ± 14	**0.04**	−32 ± 10	−25 ± 13	**<0.001**
PI (°)	57 ± 11	-	-	52 ± 11	-	-
SS (°)	−42 ± 10	−42 ± 9	0.9	−43 ± 9	−39 ± 9	**0.02**
PT (°)	15 ± 6	15 ± 5	0.95	9 ± 3	11 ± 5	0.3

**Table 4:** Ratio of increase in height for total, thoracic, and lumbar heights with respect to the thoracolumbar length from the neutral (N) to trunk self-elongation (S) positions.

Ratio	Non-Athlete	Gymnast
Ratio of thoracolumbar spine length variation (%)	1.6 ± 0.7	1.7 ± 0.8
Ratio of thoracic spine length variation (%)	1.2 ± 0.6	1.1 ± 0.7
Ratio of lumbar spine length variation (%)	0.2 ± 0.3	0.6 ± 0.6

## Data Availability

Data are available upon request and with a justification of the research objective from the corresponding author.
